# Case Report: Technical description and clinical evaluation of three cases of unilateral biportal endoscopic decompression for symptomatic spinal epidural lipomatosis

**DOI:** 10.3389/fsurg.2024.1309202

**Published:** 2024-03-12

**Authors:** Zhengqiang Liu, Huili Cai, Hongwei Zhao, Lei Tang, Siyu Jia, Zhenyu Zhou, Can Zhuo, Haidan Chen

**Affiliations:** ^1^ Department of Spinal Surgery, The First College of Clinical Medical Science, China Three Gorges University & Yichang Central People's Hospital, Yichang, Hubei, China; ^2^ Department of Hematology, The First College of Clinical Medical Science, China Three Gorges University & Yichang Central People's Hospital, Yichang, Hubei, China; ^3^ Graduate School, Ningxia Medical University, Yinchuan, China

**Keywords:** spinal epidural lipomatosis, unilateral biportal endoscopy, percutaneous biportal endoscopic surgery, spinal decompression, SEL grading

## Abstract

**Objective:**

To investigate the clinical characteristics and outcomes of three patients with symptomatic Spinal epidural lipomatosis (SEL) treated using Unilateral Biportal Endoscopic (UBE) surgery.

**Methods:**

This report retrospectively analyzed the clinical data of three patients with SEL admitted to our hospital. The analysis covers onset characteristics, clinical manifestations, and the most recent radiologic grading system of neural compression (Manjila classification). Furthermore, it details the decompression accomplished through the application of a minimally invasive UBE surgical technique, specifically targeting the removal of proliferated fat responsible for nerve and spinal cord compression.

**Results:**

This technique was performed successfully in 3 patients with SEL. Radiating pain was reduced, and the functional disability and radiologic compression were improved in all three patients. Postoperative spinal instability and surgical complications related to the procedure were not observed.

**Conclusions:**

For SEL, timely diagnosis and appropriate intervention can prevent the progression of neurological disability. UBE is a minimally invasive muscle-preserving technique that achieves neural decompression directly by the removal of excessive intraspinal adipose tissue buildup.

## 1 Introduction

**Figure 1:**
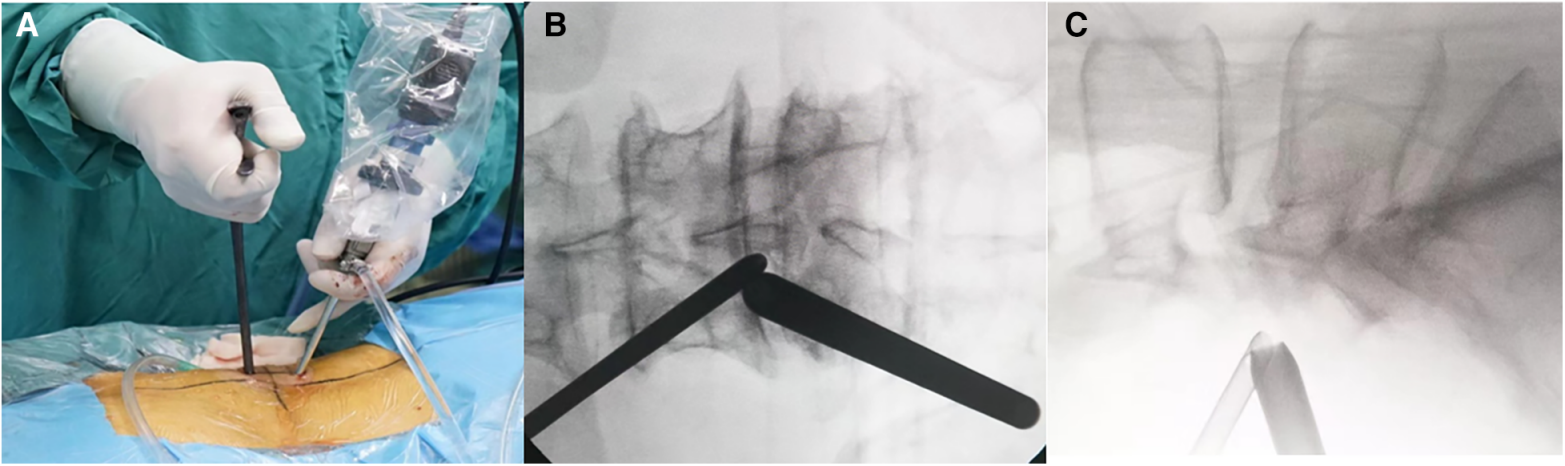
(**A**) Surgeons performing UBE surgery for treating SEL. (**B**–**C**) The positions of the viewing and working portals were confirmed using fluoroscopy in both anteroposterior and lateral views.

Spinal epidural lipomatosis (SEL) is characterized by an abnormal accumulation of encapsulated adipose tissue within the spinal epidural space. Asymptomatic SEL is usually detected only during a radiologic examination, while some symptomatic SEL can cause varying degrees of back pain, myelo-radiculopathy or cauda, which can severely affect patients' quality of life. Due to the prevalence of other coexisting degenerative that can cause similar neurological compression symptoms, the authors believe that using imaging alone to diagnose SEL as symptomatic, without clinically evaluating the extent of degenerative spine disease is highly highly erroneous. Magnetic resonance imaging is still acknowledged as the gold standard investigation to assess the extent and severity of SEL. Although the optimum method of treatment for SEL has not yet been established, surgical decompression to remove excess fatty tissue is a reasonable option for patients with acute cord compression, cauda equina syndrome (CES), or for those who have failed conservative management. Unilateral biportal endoscopy (UBE) is a novel minimally invasive spine surgery technique that has been used to treat degenerative diseases such as spinal stenosis and herniated lumbar discs. This procedure offers several advantages, including a better surgical field with less tissue detection and muscle retraction, thus offering a quicker (1). Nevertheless, only one study reported the use of UBE for the treatment of SEL in three patients. To confirm the effectiveness of UBE as a treatment for SEL in different countries and regions, multicenter studies are needed. In this study, we report the first on the successful treatment of 3 SEL patients with UBE in China (Figure 1). We retrospectively analyzed the clinical data, including the etiology, clinical manifestations, diagnosis, and treatment outcome.

## 2 Operative techniques

The patient was placed in the prone position, and lesions in the intervertebral space were located using a Kocher clamp under fluoroscopy. After adequate anesthesia was obtained, and patient positioned prone, routine sterile preparation of the surgical field was performed. Intraoperative neurophysiologic function testing was conducted utilizing a Cascade PRO intraoperative monitor (Cadwell Laboratories Inc., Kennewick, WA, USA). Two 0.9 cm skin incisions were made between the spinous processes. The endoscope was placed in the upper portal, and surgical tools were placed in the lower portal. First, dilators were used to split the paraspinal muscles, and soft tissue was detached from the interlaminar space. The position of the intervertebral space was confirmed using C-arm fluoroscopy. The working channel and surgical field of view were adjusted. Using a radiofrequency device, the lower margin of the upper lamina and the upper margin of the lower lamina were dissected, and ipsilateral pedicle-to-pedicle decompression was performed. Next, hypertrophic ligamentum flavum was removed to relieve pressure on the spinal cord. Subsequently, Kerrison punch and pituitary forceps were used to carefully clean the epidural fat and separate the nerve root while monitoring the nerve roots effectively. At the end of the operation, we confirmed pulsation of the thecal sac and decompression of bilateral traversing nerve roots. After successulfully acheiving hemostasis, and the area was repeatedly flushed for to check for any other bleed. The endoscope and cannula were removed, and the skin was sutured.

## 3 Case presentation

The clinical features of the 3 patients were summarized in Table 1.

**Table 1:** The clinical features of the 3 patients were summarized 4.29E-05.

	Patient 1	Patient 2	Patient 3
Gender	Male	Male	Male
Duration of symptoms before surgery (years)	0.5	10	0.5
BMI at time of neurological	25.8	26.6	31.5
Comorbidities	Hypertension, multiple kidney stones and renal cyst	Hypertension	Hypertension, interstitial pneumonia, peripheral neuropathy
SEL grading at the treated levels			
Manjila grading (2)	Type II moderate	Type II moderate	Type II moderate
Lee grading (3)	2	3	2
Ishikawa grading (4)	3	3	2
Postoperative follow-up			
Duration of follow up	1.5 year	1.5 year	2.5 years
Neurological status at last follow-up	Full recovery	Full recovery	Full recovery

### 3.1 Patient 1

A 66-year-old male with a BMI of 25.8. Six months ago, gradually developed low back pain, radiating to hip and lateral side of both legs. The pain worsened after prolonged standing, walking and working and relieved when bending and crouching. 1 week ago, his walking was limited to approximately 20 meters due to pain. On admission, his pain level measured on the visual analog scale (VAS) was 8, and his functional disability assessed by the Oswestry Disability Index (ODI) was 74. Relevant history included hypertension for 10 years as well as multiple renal cysts and stones.

**Figure 2:**
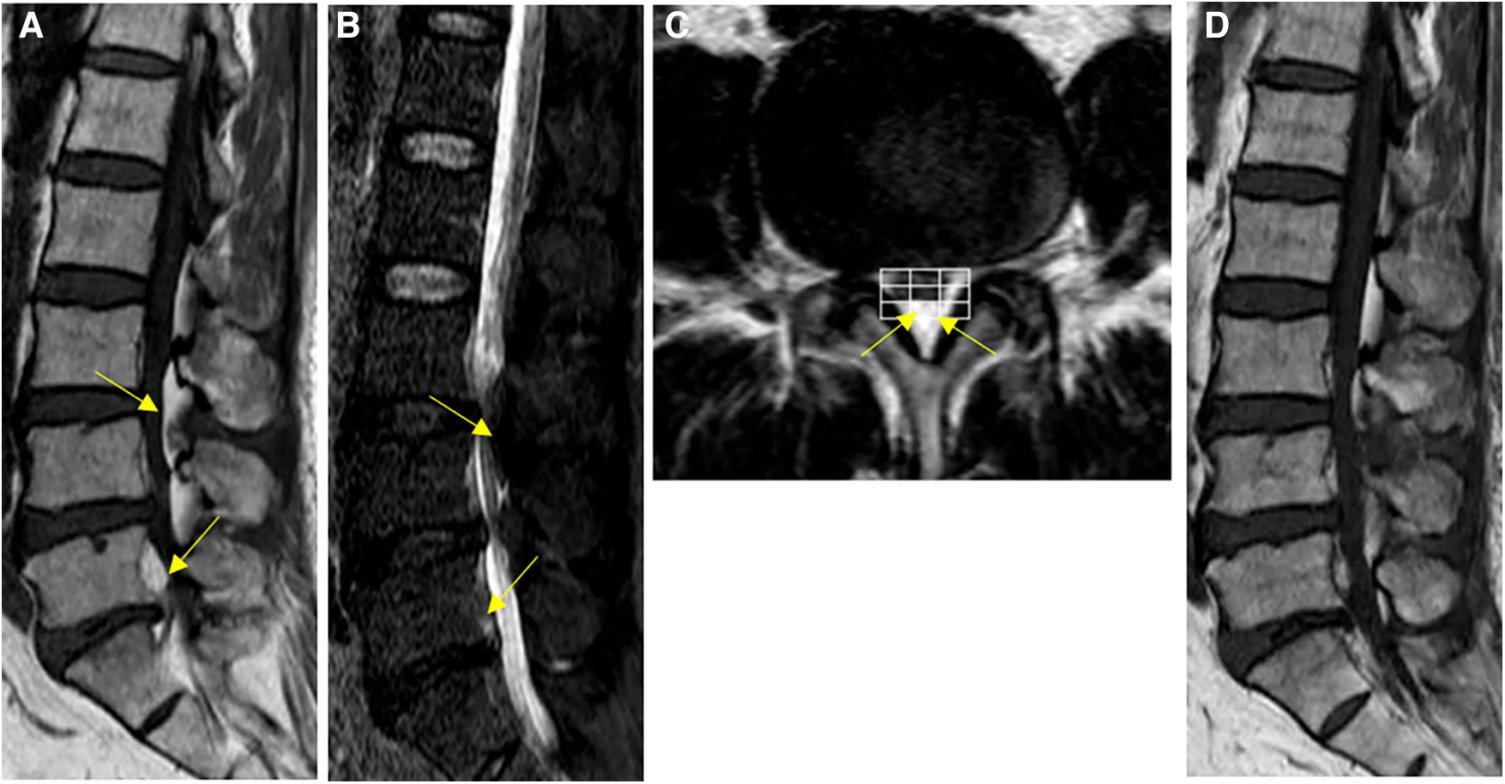
Spinal MRI images of patient #1. (**A**–**C**) Preoperative Sagittal T1, STIR and axial T2 weighted images show an overgrowth of epidural fat compressing the dural sac at L3–L5 (arrows), Manjila grading as Type II (dorsal) moderate severity with ventrally pushed thecal sac. Of note, L5-S1 level showed Manjila Type I (ventral) SEL with significant severity with dorsally pushed thecal sac. (**D**) Postoperative T1 weighted images showed reduction of SEL at the operated levels with focally diminished dorsal compression of thecal sac from the fatty tissue.

The patient presented with MRI revealing hyperintense T1 and T2 extradural signal from L3-L4 and L4-L5. T2 Fat suppression image shows suppression of adipose tissue, Manjila grading was done and it showed type II (dorsal) moderate severity (Figures 2A–C). Patient underwent a lumbar UBE decompression from L3 to L4 with excision of the intraspinal epidural fat (Figure 2D). Postoperatively, patient reported relief of lower extremity numbness and pain. The patient was discharged on day 7. On follow up, semi-annual phone calls were made, and no symptoms recurred even at 1.5 years of follow up. At the last follow-up, the VAS score improved to 1, indicating a significant reduction in pain, and the ODI improved to 8.

### 3.2 Patient 2

A 66-year-old male with a BMI of 26.6 had been experiencing back pain for 10 years. In the past 10 months, the patient's back pain worsened, accompanied by left lower limb pain that worsens after exercise. Physical examination revealed left lower limb weakness and tenderness along the course of the left sciatic nerve. The straight leg raising test was positive. On admission, his pain level measured on the VAS was 39, and his functional disability assessed by the ODI was 39. Relevant history included hypertension for 2 years.

**Figure 3:**
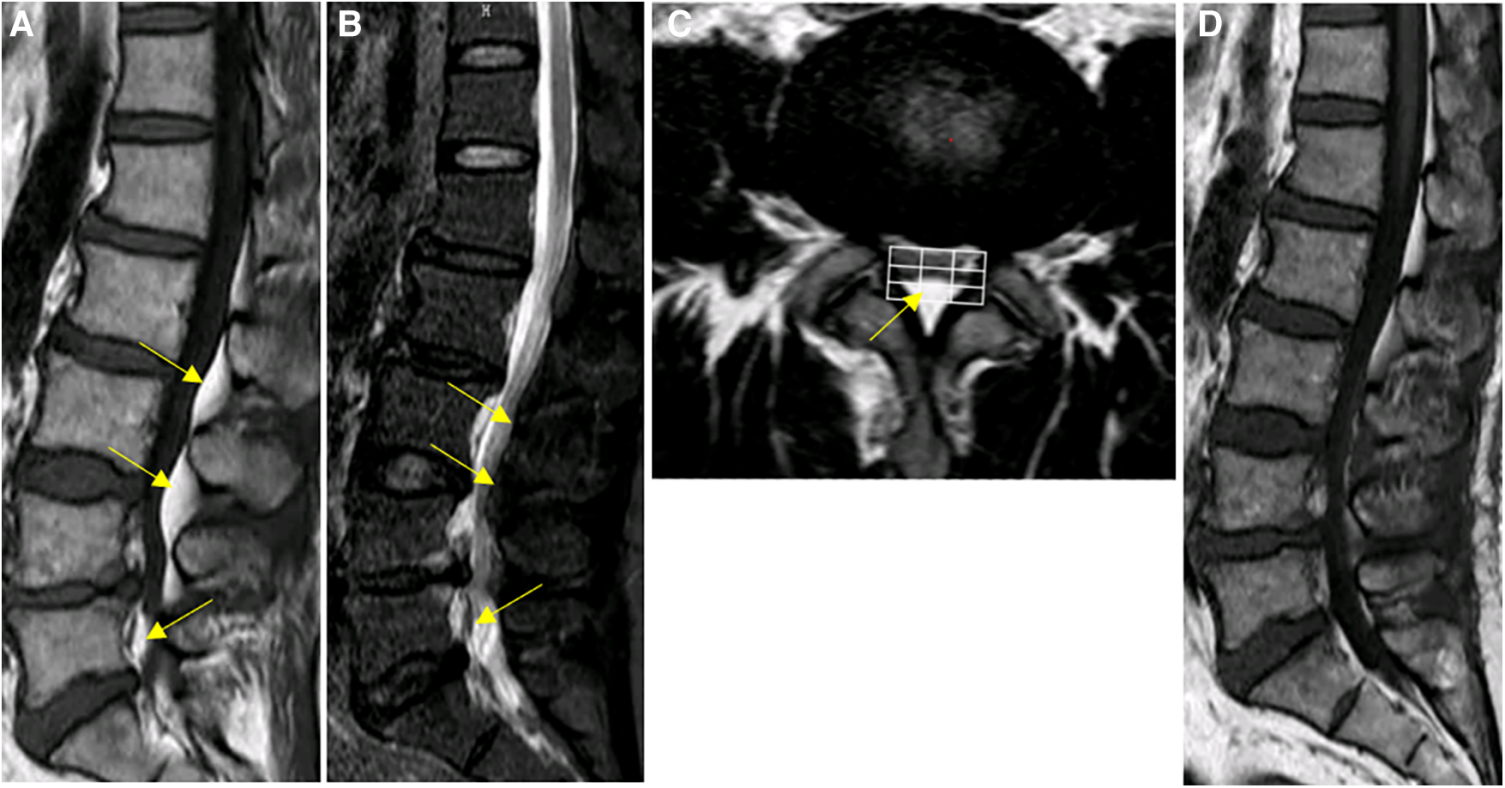
Spinal MRI images of patient #2. (**A**–**C**) Preoperative Sagittal T1, STIR and axial T2 weighted images show an overgrowth of epidural fat compressing the dural sac at L2–L5 (arrows), Manjila grading as type II (dorsal) moderate severity. Notably, the L5-S1 levels demonstrated Manjila Type III (concentric) type mild SEL. (**D**) Postoperative T2 weighted images show Reduction of L3-L5 spinal stenosis.

The patient presented with MRI revealing hyperintense T1 and T2 extradural signal fromL2-L5. T2 Fat suppression image showed suppression of adipose tissue, Manjila grading as type II (dorsal) moderate severity (Figures 3A–C). Patient underwent a lumbar UBE decompression from L3 to L5 with excision of the intraspinal epidural fat (Figure 3D). Postoperatively, patient reported relief of lower extremity numbness and pain. The patient was discharged on day 8. The patient's recovery was seen with MRI at the outpatient clinic three months post-surgery which was favorable should be replaced with remarkable. During the 1.5-year follow-up, up. With semi-annual phone calls and her lower extremity symptoms had completely resolved. At the last follow-up, the VAS score improved to 1, and the ODI improved to 4.

### 3.3 Patient 3

A 67-year-old male with a BMI of 31.5. The patient developed pain and numbness in both lower extremities without apparent cause 6 months ago, aggravated by activity and accompanied by intermittent claudication. On physical exam, the patient showed pressure pain in the spinous process near L4 and bilateral postero-lateral lower extremities, L5-S2 bilateral hypoaesthesia. On admission, his pain level measured on the VAS was 7, and his functional disability assessed by the ODI was 52. Relevant history included hypertension for 5 years and Abnormal electromyography, consider peripheral neuropathy.

**Figure 4:**
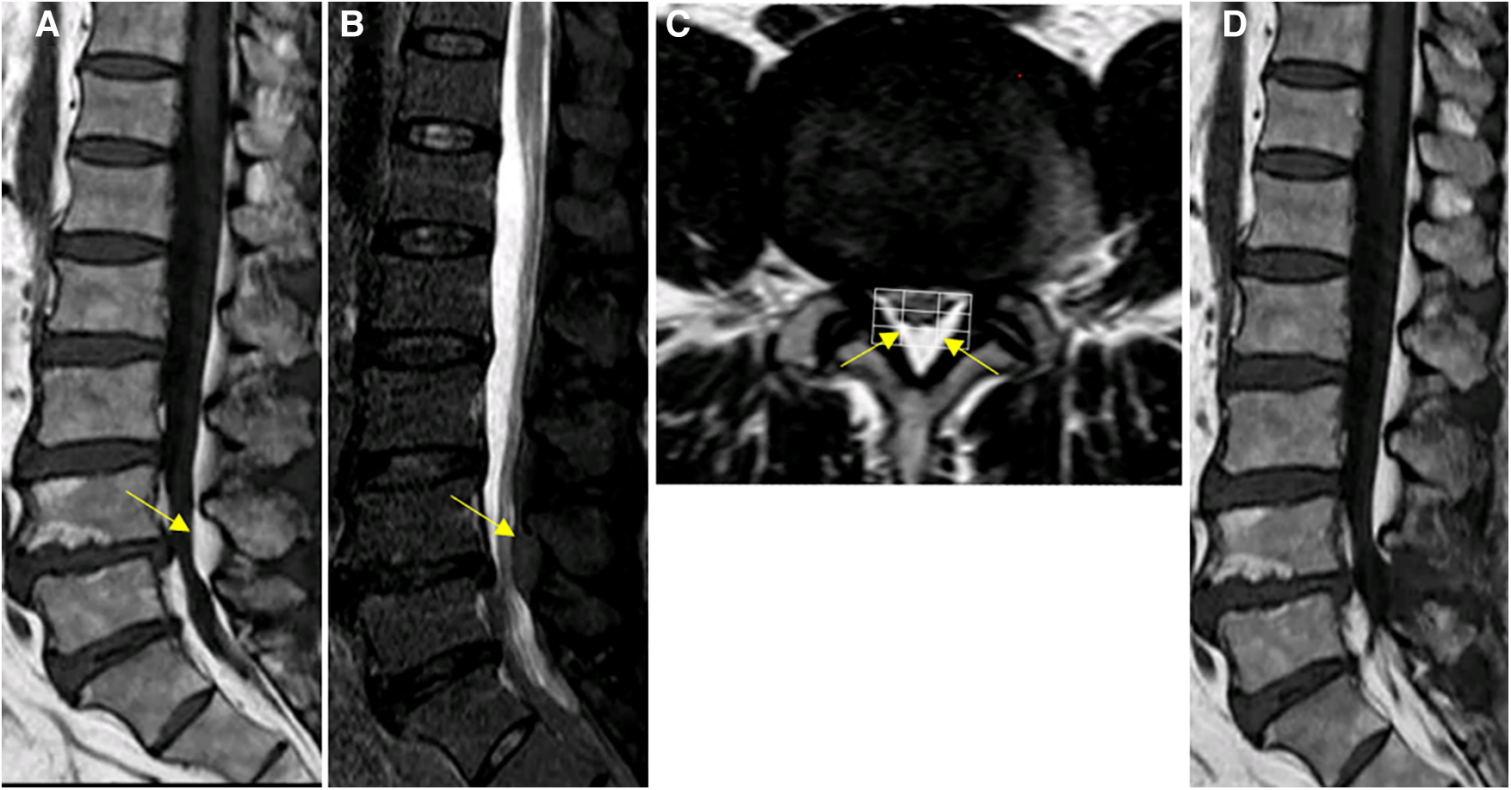
Spinal MRI images of patient #3. (**A–C**) Preoperative Sagittal T1, STIR and axial T2 weighted images show an overgrowth of epidural fat compressing the dural sac at L4–L5 (arrows), Manjila grading as type II (dorsal) moderate severity and Manjila Type III (concentric) moderate SEL seen caudally. (**D**) Postoperative T2 weighted images show reduction of L4-L5 spinal stenosis.

Sagittal MRI image showed hyperintense T1 and T2 extradural signal from L4–L5. T2 Fat suppression image showed suppression of adipose tissue, Manjila grading as type II (dorsal) moderate severity (Figures 4A–C). Patient underwent a lumbar UBE decompression from L4 to L5 with excision of the intraspinal epidural fat (Figure 4D). Postoperatively, patient reported immediate resolution of lower extremity numbness and pain. The patient was discharged on day 5. The patient's recovery seen with MRI at the outpatient clinic three months post-surgery was favorable. During the 1.5-year follow-up, semi-annual phone calls were made, and no symptoms recurred. At the last follow-up, the VAS score improved to 1, indicating a significant reduction in pain, and the ODI improved to 4.

## 4 Discussion

SEL is marked by the abnormal deposition of fatty tissue within the epidural space of the spinal canal. Such accumulation has the potential to cause progressive compression on the spinal cord or nerve roots (5). SEL primarily affects middle-aged and older men. The pathogenesis of the condition remains unclear but may be caused by factors such as exogenous steroid use, endogenous steroid hormonal disease, obesity, surgically induced and idiopathic disease among others (6). Exogenous steroid use is generally accepted as the most common cause of SEL, and it is regarded as the most significant risk factor for developing SEL. Fogel et al. reported the exogenous steroid group represents 55.3% of cases (7). In 2008, Al-Khawaja et al. analyzed a total of 111 patients with SEL and found that more than 50% of cases were due to exogenous steroid use (8). The link between obesity and SEL, however, is not without controversy. Aliciglu et al. found that the thickness of epidural adipose tissue did not significantly correlate with BMI or waist circumference (9). Meanwhile Yildirim et al. conducted an analysis of 199 SEL patients and suggested that in this study population, with every 1-point increase in BMI, the likelihood of developing SEL increases by 13% (10). The three male patients in our case series had no clear history of exogenous steroid hormone intake or endogenous steroid hormone disease. However, all three patients were overweight (BMI > 25), with one meeting the criteria for obesity (BMI > 30). We attributed their conditions to SEL induced by obesity.

SEL has traditionally been characterized as a rare medical condition. However, recent studies challenge this perception. Malone et al. conducted a retrospective analysis of MRI images from 831 patients diagnosed with spinal stenosis, revealing SEL in 6.26% of cases (11). In another large series of lumbosacral MRI with 450 patients, a prevalence of 16.7% was observed; however, there is only an 8% reporting rate (12). On one hand, the rise in obesity rates worldwide over the decades may contribute to the gradual increase in the prevalence of SEL. On the other hand, the lack of attention from radiologists and surgeons might lead to under diagnosis, making SEL easily overlooked.

Patients with symptomatic SEL may experience various symptoms, such as radiculopathy, myelopathy, claudication, cauda equina syndrome and paraparesis/plegia. These symptoms arise due to compression caused by the accumulation of excess fatty tissue in the epidural space, and the exact presentation depends on the location and degree of compression (13). Magnetic resonance imaging scans are considered the gold standard in evaluating adipose tissue in SEL (14). Borre et al. initially introduced the SEL grading system, utilizing MRI for assessment (15). Grading relies on the proportion or percentage of the spinal canal diameter occupied by epidural fat. Grade I ranges from 41% to 50%; grade II from 51% to 74%; and grade III equal to or greater than 75%. However, the Borre grading system, relying solely on cross-sectional assessment and it falls short in comprehensively evaluating severe cases with SEL spanning multiple spinal segments. The grading system proposed by Ishikawa et al. assesses both sagittal and cross-sectional MRI images (4).

However, our experience with three symptomatic SEL cases suggests that the aforementioned older SEL grading systems described are highly demanding in terms of the expertise required by radiologists and surgeons. Consequently, we employed the recently introduced Manjila grading to analyze these three cases (2). This classification system employs a 3 × 3 grid, providing a straightforward and intuitive method for grading segmental SEL severity at all levels, except L5-S1 levels, where a 3 × 1 grid was employed to assess the severity of SEL. The Manjila classification template can also be utilized in machine learning algorithms for the radiological grading of SEL, making it easily interpretable by artificial intelligence solutions. This novel classification offers precise documentation of (a) the extent and type of SEL in sagittal MR images and (b) severity of SEL at each level on axial MR images (the slice of the latter being dictated by the worst level of stenosis noted on sagittal view to save the reporting time) described in a segmental numerical manner pertaining to each vertebral body. This radiologic grading makes the inter-disciplinary communication easier and reliable between radiologists and treating surgeons/pain physicians.

Importantly a recent update of the MRI grading for spinal stenosis included SEL as one of the three main causes of lumbosacral spinal stenosis (along with disc pathology, ligamentous thickening/hypertrophy, and facet joint arthritis with or without synovial cysts) indicated as a subcategory Alpha in Manjila grading of SEL. This gives a complete radiological picture of coexisting degenerative spine disease in conjunction with SEL (16). This suggests that SEL is gaining prominence in the awareness of clinicians and radiologists. As the diagnosis of SEL advances in the population, the aggressive quest of effective treatments becomes relevant.

The treatment approach for SEL can vary from person to person. Fogel et al. (7). conducted a study and reported a 66.7% success rate in patients with SEL who underwent laminectomy and debridement, compared to 81.8% success rate in patients treated conservatively with a strict weight loss regimen. This indicates that there is still debate on whether conservative treatment is preferable. In cases where conservative treatment is chosen, if the use of external steroids is the underlying cause of SEL, it should be reduced or discontinued under the guidance of an endocrinologist. For obese patients, an endocrine evaluation is necessary to rule out the presence of endogenous steroid diseases. Weight loss can help reduce epidural fat (17), and bariatric surgery is also a viable option (18). When patients fail conservative treatment or develop acute and severe symptoms of nerve compression, surgical decompression is required. Advances in endoscopic spine surgery techniques have been robustly pursued across the world. Conditions such as disc degeneration, central lumbar stenosis, lumbar foraminal stenosis, and lumbar spondylolisthesis are all now amenable to endoscopic treatment (19). Yu et al. (20) documented the successful treatment of a patient with symptomatic SEL through percutaneous full-endoscopic uniportal decompression.

In this study, we present the application of UBE for the treatment of SEL. In UBE, two portals are created on the same side; one is for the optical instrument and irrigation system, and the other is for the surgical instrument used to perform decompression or discectomy. Based on our surgical experience, we have identified several advantages of utilizing UBE for the treatment of SEL requiring surgical intervention: (1) Patients with SEL often have a higher BMI, and UBE is not hindered by the challenges of obesity when creating longer working channels. (2) UBE utilizes the longer working pipeline, allowing for better access and maneuverability during the procedure. (3) UBE provides clear visualization of neural elements, surrounding soft tissues, vascular structures, and bony structures. This creates an optimal environment for delicate nerve manipulation and a safe decompression process (21). (4) Additionally, UBE makes it easier and safer to manipulate bone and remove the ligamentum flavum, facilitating the removal of excess fatty tissue.

To validate the applicability of UBE for the treatment of SEL in various regions with different economic and technological conditions, it is important to conduct multicenter studies with large sample sizes. As of our knowledge, there is only one report by a South Korean scholar discussing the use of UBE in treating three patients with SEL (22). However, due to the low prevalence of SEL and the ongoing controversy surrounding its optimal treatment, it becomes even more crucial to report relevant studies conducted in hospitals across different regions. These reports will serve as valuable contributions and facilitate the analysis of larger sample sizes in the future. Our study contributes additional favorable evidence supporting the use of UBE in SEL treatment. Specifically, our findings demonstrate that all three patients treated with UBE showed improvement in postoperative neurological symptoms and experienced no significant complications during the final follow-up.

However, it is important to acknowledge the limitations of our report. One limitation is the small sample size. Only six SEL patients, including the three in our study, have undergone UBE. To obtain more robust and reliable results, a larger multicenter prospective study that follows patients over a longer period of time is necessary to confirm the effectiveness of UBE in treating SEL. Additionally, the low prevalence of SEL poses challenges in conducting randomized controlled trials to compare the effectiveness of surgical and non-surgical treatments. Gathering a sufficient number of participants for such studies can be difficult due to the rarity of the condition. Therefore, more research is needed to explore the optimal treatment approaches for SEL.

## 5 Conclusion

In conclusion, while our findings suggest the potential benefits of UBE as a surgical modality for SEL patients, further studies with larger sample sizes and longer follow-up periods are required to validate our results and establish more definitive conclusions regarding the efficacy of different treatment options for SEL.

## Data Availability

The original contributions presented in the study are included in the article/Supplementary Material, further inquiries can be directed to the corresponding author.
